# Vertically Coupled Plasmonic Racetrack Ring Resonator for Biosensor Applications

**DOI:** 10.3390/s20010203

**Published:** 2019-12-30

**Authors:** Kirill V. Voronin, Yury V. Stebunov, Artem A. Voronov, Aleksey V. Arsenin, Valentyn S. Volkov

**Affiliations:** 1 Center for Photonics & 2D Materials, Moscow Institute of Physics and Technology, 9 Institutsky Lane, Dolgoprudny 141700, Russia; voronin.kv@phystech.edu (K.V.V.); stebunov@phystech.edu (Y.V.S.); voronov.artem@gmail.com (A.A.V.); arsenin.av@mipt.ru (A.V.A.); 2 Skolkovo Institute of Science and Technology, Bolshoy Boulevard 30, bld. 1, Moscow 121205, Russia; 3 GrapheneTek, 7 Nobel Street, Skolkovo Innovation Center, Moscow 143026, Russia

**Keywords:** biosensors, plasmonic ring resonators, metal-strip waveguides

## Abstract

Plasmonic chemical and biological sensors offer significant advantages such as really compact sizes and extremely high sensitivity. Biosensors based on plasmonic waveguides and resonators are some of the most attractive candidates for mobile and wearable devices. However, high losses in the metal and complicated schemes for practical implementation make it challenging to find the optimal configuration of a compact plasmon biosensor. Here, we propose a novel plasmonic refractive index sensor based on a metal strip waveguide placed under a waveguide-based racetrack ring resonator made of the same metal. This scheme guarantees effective coupling between the waveguide and resonator and low loss light transmittance through the long-range waveguide. The proposed device can be easily fabricated (e.g., using optical lithography) and integrated with materials like graphene oxide for providing adsorption of the biomolecules on the sensitive part of the optical elements. To analyze the properties of the designed sensing system, we performed numerical simulations along with some analytical estimations. There is one other interesting general feature of this sensing scheme that is worth pointing out before looking at its details. The sensitivity of the considered device can be significantly increased by surrounding the resonator with media of slightly different refractive indices, which allows sensitivity to reach a value of more than 1 μm per refractive index unit.

## 1. Introduction

In recent years, researchers proposed various schemes of optical label-free biosensors, which simultaneously have high sensitivity and a small footprint, allowing their integration into electronic devices [1,2]. The operation principle of most optical biosensors is based on the detection of changes in optical properties induced by the adsorption of analyzed substances on the surface of optical structures. An efficient way to track changes in refractive index (RI) is by using photonic micro-ring resonators [3,4,5,6,7,8,9,10] or interferometers [11,12] due to the strong dependence of their optical resonance conditions on the optical properties of the surrounding medium. The development of plasmonics significantly influenced modern biosensing technology and, currently, most commercial optical biosensors are based on surface plasmon resonance (SPR) excited using the Kretschmann configuration [13,14,15,16,17,18].

The other interesting approach in plasmonic biosensing technology is based on the same principles as those used in integrated nanophotonic biosensors. For example, various schemes with plasmon microresonators were already proposed and discussed [19,20,21,22,23,24]. This scheme has some advantages over SPR biosensors that are commonly used today; it is much more compact and easier to integrate into digital devices. The simplicity of these schemes is that they do not contain any moving parts, such as rotating prisms in SPR systems, except for a tunable small range laser.

In this paper, we present a compact RI sensor based on an integrated strip-waveguide-based plasmonic racetrack ring resonator (this form is usually used for better coupling with the waveguide [25,26,27]) coupled with a plasmonic strip waveguide. The coupling between the eigenmodes of the strip waveguide and the resonator leads to the appearance of minima and maxima in transmission spectra. The change in the RI of an external medium causes a wavelength shift of resonant peaks. The performance of the proposed sensor is investigated using analytical calculations and numerical simulations in the COMSOL Multiphysics software package, which includes calculations of the biosensing sensitivity and quality factor of the resonator. Despite the fact that plasmonic resonators have a much lower Q-factor compared to photonic ones with few exceptions available [28,29], a sensitivity of about 1.2 μm/refractive index unit (RIU) can be achieved for a biosensor based on a plasmonic micro-ring resonator, whereas the typical sensitivity of biosensors based on photonic resonators does not exceed 0.5 μm/RIU.

## 2. Device Configuration and Operation Principles

**Figure 1:**
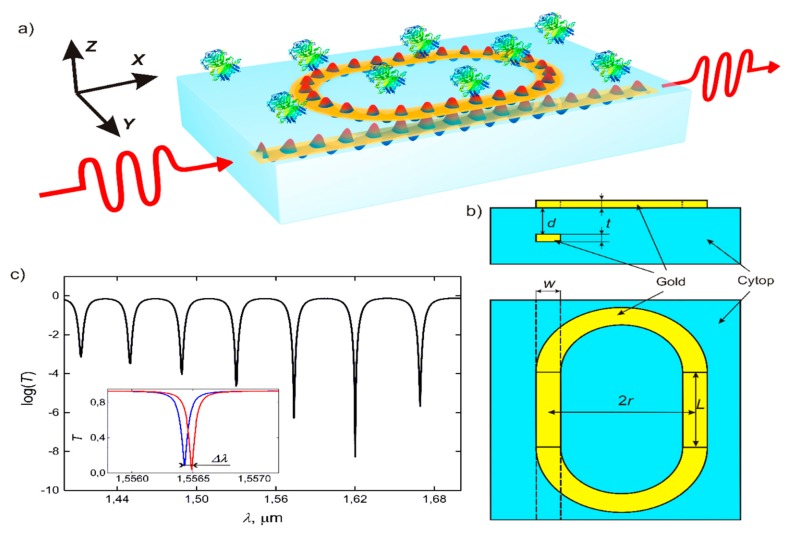
Scheme of the biosensor: (**a**,**b**) the long-range surface plasmon polariton (LRSPP) strip waveguide is located inside the dielectric. The resonator, which is the same waveguide closed in a ring, is located on top of the substrate, on the boundary with the test solution. (**c**) Dependence of logarithm of transmission through the system from the wavelength of the input signal. The shift of resonance curve that appears when the refractive index (RI) of the test solution above the resonator changes by 5 × 10^−5^.

The functional element of the proposed plasmonic integrated biosensors is a resonantly transmitted system composed of a vertically coupled plasmonic waveguide and racetrack ring resonator (Figure 1). The plasmonic waveguide embedded into the symmetric dielectric media is a thin strip of metal, which supports two plasmonic modes with symmetric (S) and antisymmetric (AS) distributions of the *E_z_* component of exponentially decaying electromagnetic fields [30]. The S-mode corresponds to an antisymmetric distribution of the *E_x_* component, which is called long-range plasmon, and the AS-mode corresponds to an antisymmetric distribution of the *E_x_* component, which is called short-range plasmon. The long-range surface plasmon polariton (LRSPP) mode gets less confined within the metal (as the metal thickness decreases) and evolves into the transverse electromagnetic TEM mode of the surrounding dielectric media. The latter results in a longer decay length into the surrounding dielectric media and a longer propagation distance, due to a decrease in ohmic losses. Oppositely, the short-range mode gets more concentrated within the metal and, for this reason, is often termed as short-range SPP (SRSPP).

Above this waveguide, we placed a ring made of the same metal strip that represents a plasmonic resonator. If the distance between the waveguide and ring resonator is comparable or lower than the decaying length of the corresponding LRSPP waveguide mode, the waveguide becomes evanescently coupled to the resonator [31]. Practically, these schemes can be realized using a standard planar fabrication method [32]. As materials for the plasmonic waveguide, gold, silver, copper, and indium tin oxide ITO were extensively studied [33,34,35]. Here, we focus mostly on gold as the standard plasmonic material [36,37,38]. We mostly consider a long-range mode since extremely high propagation losses in the short-range mode make the observation of optical resonances in the transmission spectra difficult. For the existence of the LRSPP, media above and under the resonator should have close refractive indexes. Consequently, we need to choose a material with RI close to the RI of water in a flow cell. The symmetric dielectric media can be realized using thin films of optically transmitted polymers such as CYTOP [39], which were demonstrated for the fabrication of long-range plasmonic waveguides and various schemes with them [40]. The refractive index of further considered CYTOP is 1.33 at 1550 nm. Unless otherwise specified, the geometrical parameters of the proposed scheme are as follows: the thickness of the metal layer *t* = 40 nm, the strip width *w* = 5 μm, the distance between the waveguide and the resonator *d* = 6 μm, the radius of the resonator *R* = 50 μm, and the length of the straight part of the resonator *L* = 50 μm.

The following equation defines the transmission of optical radiation through the optical scheme of the plasmonic strip waveguide coupled with the plasmonic micro-ring resonator [6]:

(1) $$T={\left|\frac{\cos\left(CL\right)-e^{-\gamma l}e^{i\varphi }}{1-e^{-\gamma l}e^{i\varphi }\cos(CL)}\right|}^{2},$$

where *C* is a coupling constant, *L* is the length of the coupling region, *l* is the circumferential length of the resonator, *γ* = Im*β*, *ϕ* = Re*βl*, and *β* is the propagation constant in the resonator. Figure 1 presents the dependence of the transmission on the wavelength, which is actually a sequence of resonances with the distance between them defined by

(2) $$\begin{array}{l} \frac{2\pi }{\lambda }n_{eff}(2\pi r+2L)\approx 2\pi k,\\ k\in \mathbb{Z} \end{array},$$

where *r* is the radius of circular parts of the resonator, and *n_eff_* is the effective mode index. When the RI of the media above the microresonator changes, the graph shifts by a certain distance, enabling the determination of this RI change and detection of various chemical and biological species. As shown in Figure 1, the depth of resonances in the output spectrum is not the same and varies with the wavelength because of the dependence of the coupling constant on the wavelength. The values of optical transmission at resonances lie on the curve given by the following equation:

(3) $$T_{0}=\cos\left(C(\lambda ,n)L\right).$$

Thus, the design of the optical scheme should ensure that the minimum of the curve in Equation (3) lies within the operating wavelength range, which in turn provides maximum sensitivity in the detection of RI changes.

## 3. Simulation of a Resonator and Waveguide Coupling

### 3.1. Waveguide

**Figure 2:**
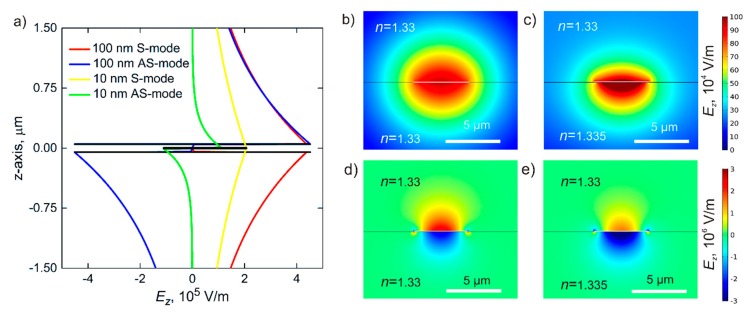
(**a**) Distribution of the field in plasmonic strip waveguide modes. Transverse electric field distributions for symmetric (red line) and antisymmetric (blue line) modes of a 100-nm waveguide and for antisymmetric (green line) and symmetric (yellow line) modes of a 10-nm waveguide with infinite width. Transverse electric field distributions for two eigenmodes of plasmonic strip 40-nm waveguides: (**b**) symmetric and (**d**) asymmetric mode in uniform medium, and (**c**,**e**) perturbation of these two modes, respectively. In the case where the waveguide lies in the boundary between mediums with different RI, Δ*n* = 0.005 (scale bar, 5 μm).

The waveguide and ring resonator in the considered biosensor are both based on thin metal films, which are enclosed into symmetric dielectric media and support two surface plasmon modes: S and AS (Figure 2a). During biosensor operation, the presence of analyte molecules leads to slight asymmetry of dielectric media, which in turn results in distortion of waveguide modes (Figure 2b). However, small RI changes less than 0.01 are compatible with the existence of both modes and retain the functionality of the proposed device. The symmetric surface plasmon mode disappears for a larger discrepancy in dielectric RI indices. However, this mode is more preferable for the realization of optical biosensors because of higher propagation lengths, i.e., several millimeters compared to tens of micrometers for antisymmetric mode.

Surface plasmons in thin metal films are described by the following dispersion equation:

(4) $$e^{-2k_{1}t}=\frac{\frac{k_{1}}{\varepsilon_{1}}+\frac{k_{2}}{\varepsilon_{2}}}{\frac{k_{1}}{\varepsilon_{1}}-\frac{k_{2}}{\varepsilon_{2}}}\frac{\frac{k_{1}}{\varepsilon_{1}}+\frac{k_{3}}{\varepsilon_{3}}}{\frac{k_{1}}{\varepsilon_{1}}-\frac{k_{3}}{\varepsilon_{3}}},$$

where *k*_1_ is the *z*-component of the wavevector in the core with the permittivity equal to *ε*_1_, and *k*_2_ and *k*_3_ are wave vectors in the cladding characterized by permittivities *ε*_2_ and *ε*_3_, respectively.

**Figure 3:**
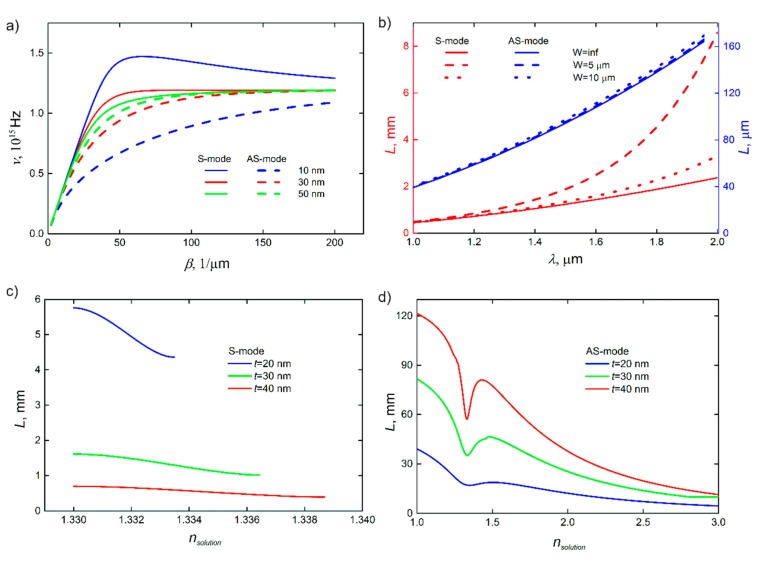
(**a**) Dispersion curves and propagation length in plasmonic strip waveguides. Relationship between frequency and propagation constant for two modes propagated in the thin metal film for different thickness of the film. (**b**) Dependence of propagation length for basic modes in strip waveguides on a wavelength with different waveguide width. (**c**,**d**) Propagation length depending on the RI of a test solution for different thicknesses of the waveguide for symmetric (S)- and antisymmetric (AS)-modes, respectively. The RI of the dielectric substrate in this scheme is 1.33.

Figure 3a shows dispersion curves obtained using Equation (4) for different thicknesses of the metal film. For thicker films, dispersion of surface plasmons tends to that for surface plasmon waves excited on the surface of bulk metal. In addition, surface plasmons in thick films show less response to the RI changes from one side of the metal films, lowering the sensitivity of biosensing. Another important quality of surface plasmons that depends on the thickness of the metal layer is the propagation length. From Equation (4), it can be represented from the imaginary part of the wavevector for S and AS modes.

(5) Lsym=−2Re(ε1)Im(ε1)ε23/2k03t2.

(6) Lantis=−(Re(ε1))3k0t28Im(ε1)ε22ε2+(2ε2ε1k0t)2.

Therefore, the biosensor operating mostly by means of the S-mode should be based on thinner films. However, weak confinement and extremely large mode size (up to 10*λ*) do not allow realizing waveguide bend with small curvature radius [41,42] and performing effective coupling between waveguide and resonator (see Section 3). Thus, in our next calculations, unless otherwise specified, we analyze optical schemes based on 40-nm-thick gold films, which can support surface plasmon modes with a propagation length of around 1 mm.

Strips made from the gold films also support both plasmonic waveguide modes, which are characterized by higher delocalization compared to modes in continuous films. Due to this, the propagation lengths of SPP in metal stripes are higher for narrower waveguides (Figure 3b). In addition, less localization of waveguiding modes in metal stripes leads to the lower stability of the mode in asymmetric media and higher scattering losses on curved parts of the scheme. In the subsequent calculations, we primarily consider the plasmonic waveguides based on 40-nm-thick gold stripes with a width of 5 μm. If such a plasmonic strip waveguide is made on CYTOP polymer, the mode exists for dielectrics at the other side with RIs from 1.32 to 1.34, which determines the operational range of the biosensor (Figure 3c,d). In this sense, thicker metal waveguides are also more preferable for the fabrication of the proposed optical biosensors because of the wider operational range for analyzed solutions.

### 3.2. Coupling of Modes

**Figure 4:**
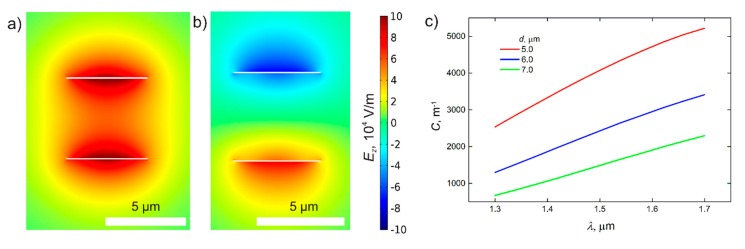
(**a**,**b**) Distribution of the *z*-component of the field in modes of the system consists of two coupled plasmonic strip waveguides. Transverse electric field distributions for two eigenmodes (perturbed S-mode of the separated waveguide in the presence of the second waveguide) of the system of coupled plasmonic waveguides with thickness *t* = 40 nm (scale bar, 5 μm). (**c**) The difference between these modes characterizes the energy transfer between waveguides. The dependence of coupling constant on wavelength for strip waveguides with the different distances between them.

Coupling of the waveguide and the resonator is realized by their vertical stacking. Next, we find the coupling constant, which describes the energy transmission between two parallel plasmonic strip waveguides. Instead of one long-range S-mode, this system supports two modes with transverse electric fields around waveguides oscillating in phase and antiphase (Figure 4a). In this case, the coupling constant is proportional to the difference in wavenumbers corresponding to these modes, i.e., $C=\;\frac{\left|k\_1-k\_2\;\right|}{2}\;\leq \;k_{0}\left(n-n_{0}\right)$, where *n* is an effective mode index and *n*_0_ is the RI of the dielectric [43]. Thus, for thicker waveguides, the coupling constant for the waveguide and resonator is larger, providing more effective detection of RI changes. The coupling constants obtained by COMSOL simulations for different plasmonic strip waveguides are represented in Figure 4c.

## 4. Sensitivity

The efficiency of the proposed optical biosensor is described by the parameter of sensitivity to RI changes, which is a measure of the system response to the change in optical properties of the analyzed media. Practically, this response is usually represented by the shift of the adsorption peaks in transmission spectra. Thus, the sensitivity to RI changes is the ratio of the resonance peak shift to the corresponding change of RI changes, which in turn can be presented as a function of wavevector derivatives,

(7) $$S=\left|\frac{d\lambda }{dn}\right|=\left|\frac{\partial T}{\partial n}{\left(\frac{\partial T}{\partial \lambda }\right)}^{-1}\right|\approx \left|\frac{\partial n_{eff}}{\partial n}{\left(\frac{\partial n_{eff}}{\partial \lambda }\right)}^{-1}\right|,$$

**Figure 5:**
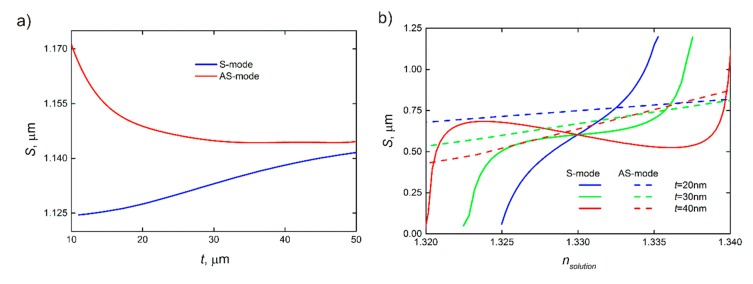
Dependence of sensitivity on essential parameters. The sensitivity of plasmonic biosensor depending (**a**) on the thickness of waveguide and type of mode, and (**b**) on RI of the test solution with different thicknesses. The RI of the dielectric substrate in this scheme is 1.33. The difference between RI of media on both sides of the waveguide has a significant effect on sensitivity.

Therefore, the sensitivity mainly depends on the thickness of the waveguide and RI of the surrounding media, because the wavevector of the guided plasmonic mode is unaffected by parameters of the system such as the length of the resonator and the distance between the waveguide and the resonator. The sensitivity to RI changes shows different dependence for S- and AS-modes, which increase and decrease, respectively, with the thickness of the gold waveguide (Figure 5a). However, for instance, for LRSPP, the total increase in the sensitivity is just about 10% for the thickness increase from 10 to 50 nm. Thus, for the sake of practical application, the waveguide thickness can be chosen considering its other impacts on the device performance.

The change in the RI of the media above the plasmonic resonator results in different dependencies of the sensitivity in both cases of LRSPP and SRSPP. As shown in Figure 5b, the corresponding change in the RI in the range of 1.32–1.34 leads to only a slight change in sensitivity. By contrast, even the existence of the S-mode is limited by the RI of the analyzed media, which is represented by the S–RI curve breaks in Figure 5b. In turn, the width of this range strongly depends on the thickness of the plasmonic resonator, and it is equal to 0.010, 0.015, and 0.020 RIU for the thicknesses of 20, 30, and 40 nm, respectively. Thus, thicker resonators are more favorable for the measurements in broader ranges of RI of the analyzed media.

Another important characteristic of optical biosensors is the detection limit (DL), which is the minimum measurable change in the RI that can be represented as follows [44]:

(8) $$DL=\frac{\delta \lambda }{S},$$

where Δ*λ* is wavelength accuracy of a used spectrometer. Considering the wavelength accuracy of 0.001 nm that is easily attainable with a commercial spectrum analyzer, we obtain the resolution of 8 × 10^−7^ RIU for the proposed biosensor. The comparison of our scheme with the most popular other schemes of biosensors is shown in Table 1. Among the compact biosensors based on interferometers and different types of photonic and plasmonic resonators, our scheme using long-range plasmonic waveguides and resonators provides better sensitivity to RI changes.

## 5. Possible Modification and Upgrades

We consider below some possible modifications of the biosensor scheme, as well as directions for further work and important points for experimental realization.

1. The scheme with two waveguides coupled to the ring resonator [48] provides more efficient biosensing when using resonators characterized by low optical losses such as photonic ones. In this case, light transmission through the device is characterized by narrower resonant peaks with zero transmission at resonance.
2. In theory, the AS-mode provides higher sensitivity than the S-mode as shown in Figure 5. However, the AS-mode propagation length is significantly less than that for the S-mode and even less than the resonator length, which makes optical biosensing highly inefficient. The situation can be different for optical biosensors exploiting plasmonic schemes based on ultrathin metal films and plasmonic two-dimensional (2D) materials [49,50,51]. As shown in Figure 5, the sensitivity to RI increases with the decreasing thickness of the plasmonic waveguide.
3. In the case of SRSPP, the intensity transmitted through the device is strongly suppressed due to absorption in the waveguide; thus, the plasmonic waveguide can be partially (except the coupling area) replaced with a photonic waveguide. To realize this, materials and geometry parameters of the dielectric waveguide should be chosen to match the propagation constant for them with the propagation constant for the plasmonic waveguide [52,53,54].

## 6. Conclusions

This paper presented a novel scheme of optical biosensors based on a vertically coupled plasmonic waveguide and racetrack ring resonator. The flow cell was placed above the resonator and used to deliver analyzed substances to the biosensor. Both the plasmonic waveguide and racetrack ring resonator were based on strips of thin metal films, which support plasmonic modes with exponentially decaying electromagnetic field in the surrounding dielectric media. Due to this, the resonator is sensitive to changes in RI of the media in the flow cell and transmits this information to the waveguide coupled to its other side. The fabrication of the proposed optical biosensors is compatible with standard microelectronic technologies and can facilitate the mass production of commercial devices. Using numerical simulations, we obtained the coupling constants for the plasmonic waveguide and resonator, as well as the parameters of plasmonic waveguide modes excited in thin gold strips. The usage of LRSPP mode in the proposed optical scheme provides better biosensing efficiency due to longer propagation length and the higher quality factor of the corresponding plasmonic resonance. Despite that, the propagation length of the LRSPP increases upon decreasing the thickness of the plasmonic waveguide, whereby gold stripes with a thickness of about 40 nm are more favorable for the fabrication of the proposed biosensors due to the higher effective index of the plasmonic mode. This allows decreasing the optical losses at the bending sections of the resonator, in addition to increasing the operational range of RI changes detectable with the proposed biosensor. The optical biosensor based on the plasmonic racetrack ring resonator supporting LRSPP mode has a sensitivity to RI changes of 1200 nm/RIU. Such high sensitivity allows reaching a DL of 8 × 10^−7^ RIU, which makes the proposed biosensor more accurate than other types of compact optical biosensors based on interferometric schemes and photonic resonators. Moreover, the considered planar structure is compatible with bioselective immobilization matrices based on two-dimensional materials [17,18], which open applications for the analysis of biochemical reactions and medical diagnostics.

## Figures and Tables

**Table 1:** Comparison of the sensitivity and detection limit (DL) for different types of biosensors. RIU—refractive index unit.

Source	Sensitivity, nm/RIU	DL
Our scheme	1200	8 × 10^−7^
Interferometer [11]	250	4 × 10^−5^
Photonic resonator biosensor [45]	200–250	5 × 10^−6^
Plasmonic resonator biosensor [46]	580	1.5 × 10^−4^
Kretschman configuration [47]	7500	10^−8^
